# Early application of metagenomics next-generation sequencing may significantly reduce unnecessary consumption of antibiotics in patients with fever of unknown origin

**DOI:** 10.1186/s12879-023-08417-3

**Published:** 2023-07-18

**Authors:** Hongmei Chen, Mingze Tang, Lemeng Yao, Di Zhang, Yubin Zhang, Yingren Zhao, Han Xia, Tianyan Chen, Jie Zheng

**Affiliations:** 1 Department of Infectious Diseases, The First Affiliated Hospital of Xi’an Jiaotong University, Xi’an, China; 2 Department of Scientific Affairs, Hugobiotech Co., Ltd, Beijing, China; 3 Department of Pharmacy, The First Affiliated Hospital of Xi’an Jiaotong University, Xi’an, China; 4 Department of Clinical Laboratory, The First Affiliated Hospital of Xi’an Jiaotong University, Xi’an, China; 5 Clinical Research Center, The First Affiliated Hospital of Xi’an Jiaotong University, Xi’an, China

**Keywords:** Metagenomic next-generation sequencing, Fever of unknown origin, Pathogen detection, Consumption of antibiotics, Pathogen spectrum

## Abstract

**Background:**

Metagenomic next-generation sequencing (mNGS) is a novel nucleic acid method for the detection of unknown and difficult pathogenic microorganisms, and its application in the etiological diagnosis of fever of unknown origin (FUO) is less reported. We aimed to comprehensively assess the value of mNGS in the etiologic diagnosis of FUO by the pathogen spectrum and diagnostic performance, and to investigate whether it is different in the time to diagnosis, length of hospitalization, antibiotic consumption and cost between FUO patients with and without early application of mNGS.

**Methods:**

A total of 149 FUO inpatients underwent both mNGS and routine pathogen detection was retrospectively analyzed. The diagnostic performance of mNGS, culture and CMTs for the final clinical diagnosis was evaluated by using sensitivity, specificity, positive predictive value, negative predictive value and total conforming rate. Patients were furtherly divided into two groups: the earlier mNGS detection group (sampling time: 0 to 3 days of the admission) and the later mNGS detection group (sampling time: after 3 days of the admission). The length of hospital stay, time spent on diagnosis, cost and consumption of antibiotics were compared between the two groups.

**Results:**

Compared with the conventional microbiological methods, mNGS detected much more species and had the higher negative predictive (67.6%) and total conforming rate (65.1%). Patients with mNGS sampled earlier had a significantly shorter time to diagnosis (6.05+/-6.23 vs. 10.5+/-6.4 days, P < 0.001) and days of hospital stay (13.7+/-20.0 vs. 30.3 +/-26.9, P < 0.001), as well as a significantly less consumption (13.3+/-7.8 vs. 19.5+/-8.0, P < 0.001) and cost (4543+/-7326 vs. 9873 +/- 9958 China Yuan [CNY], P = 0.001) of antibiotics compared with the patients sampled later.

**Conclusions:**

mNGS could significantly improve the detected pathogen spectrum, clinical conforming rate of pathogens while having good negative predictive value for ruling out infections. Early mNGS detection may shorten the diagnosis time and hospitalization days and reduce unnecessary consumption of antibiotics.

**Supplementary Information:**

The online version contains supplementary material available at 10.1186/s12879-023-08417-3.

## Background

Fever of unknown origin (FUO) is a syndrome characterized by prolonged fever with complex etiology and difficult clinical diagnosis. The definition was proposed by Petersdorf and Beesonfever as oral temperature above 38.3 °C on at least 3 occasions for more than 3 weeks, in which diagnosis could not be made after 1 week of systematic examination in an outpatient or inpatient setting [1]. There are more than 200 reported causes of FUO, about one-third of which are infectious diseases, followed by non-infectious inflammatory diseases (NIID) and tumors [2–4], with 7–51% of FUO still remained unexplained [4–6]. The difficulties in diagnosis and prolonged illness cause patients to suffer and result in high hospital and medical costs [7, 8]. Thus, more effective pathogen diagnosis techniques are needed to improve the diagnosis of FUO.

Although imaging technologies help in the localization of lesions [9–11], the development of molecular diagnostic methods provides additional opportunities for etiologic differentiation [12–14]. As a new nucleic acid test for all genomes of pathogenic microorganisms, metagenomic next-generation sequencing (mNGS) showed excellent performance for both unexpected and difficult clinical infectious diseases [15–17], and often exhibits higher diagnostic sensitivity than traditional pathogen detection methods [13]. Moreover, mNGS using cell-free DNA has been reported to help diagnose pathogens and rule out infections [18], so we hypothesized that the use of cell free mNGS may also provide useful information for FUO.

In addition, more evidences is required to determine whether mNGS should be routinely used in FUO investigations. To date, molecular methods are usually used as second-line tools for FUO [19–23], which may result in higher cost [14]. The timing and cost-effectiveness of the application of mNGS in FUO are worth discussing.

The purpose of this study was to comprehensively assess the value of mNGS in the etiologic diagnosis of FUO by the pathogen spectrum and diagnostic performance, and to investigate whether it is different in the time to diagnosis, length of hospitalization, antibiotic consumption and cost between FUO patients with and without early application of mNGS.

## Methods

### Study design

This is a retrospective cohort study. One hundred and forty-nine patients with FUO hospitalized in the Department of Infectious Disease at the First Affiliated Hospital of Xi’an Jiaotong University from January 1st, 2019 to July 30th 2021 were enrolled into the study. All of the patients were diagnosed as FUO at the admission. FUO was diagnosed based on the following criteria: oral temperature > 38.3 ℃ on at least 3 occasions (or temperature fluctuations > 1.2 ℃ within 1 day on at least 3 occasions); the diagnosis could not be made after at least 1 week of systematic and comprehensive examination in outpatient or inpatient setting. All enrolled FUO patients underwent mNGS and traditional pathogen detection methods. To ensure the homogeneity of the study population, the patients with underlying immunodeficiency were excluded due to the biologic variation among those patients, including organ-transplant recipients, patients with granulocyte deficiency and patients receiving immunosuppressive therapies.

**Fig. 1:**
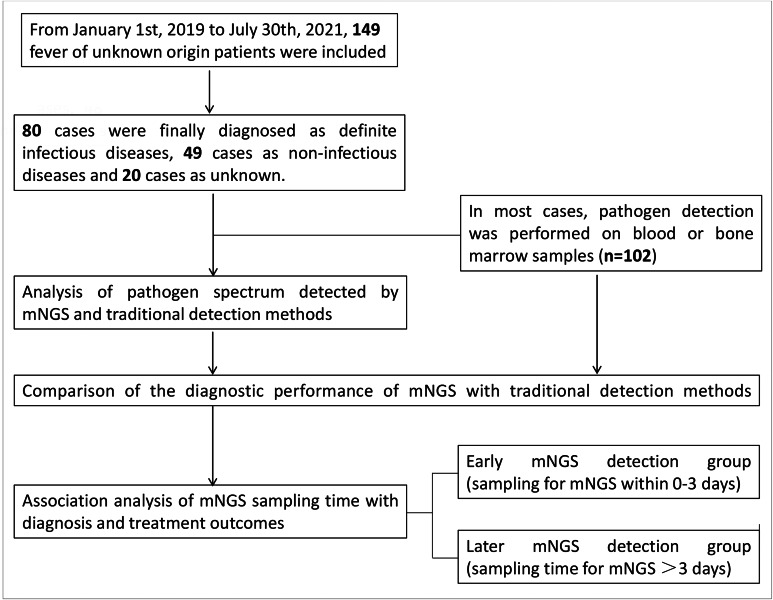
Flowchart of the study design

Samples of blood, bone marrow, bronchoalveolar lavage fluid, sputum or secretions were collected at admission. One hundred and two patients out of 149 patients had blood or bone marrow samples sampled. The type of all samples used for pathogen detection varies among FUO patients were summarized in Fig. 1, which revealed that blood and bone marrow samples (68%) accounted for the majority of the samples. The pathogen spectrum was detected by mNGS, culture and other traditional methods for each patient. The final diagnosis was evaluated for all patients by the expert group including three experienced physicians. The flow chart of the study is shown in Fig. 1.

**Fig. 2:**
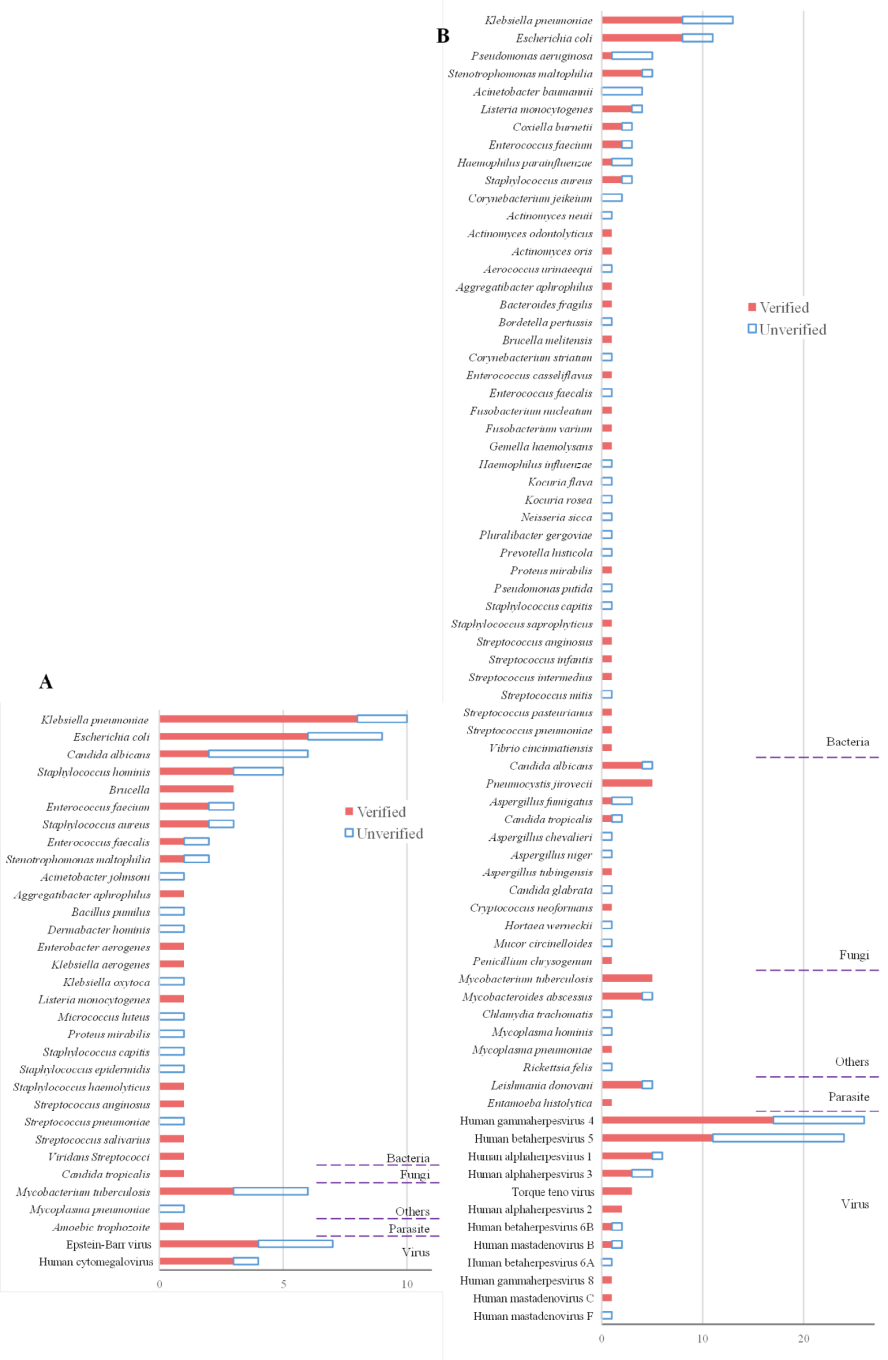
Pathogen spectrum of pathogens. **(A)** The distribution of pathogens detected by culture and CMTs. **(B)** The distribution of pathogens detected by metagenomic next generation sequencing (mNGS). Each bar is divided into two parts, indicated by different colors. Verified represents pathogens detected by traditional detection methods (culture and CMTs) or mNGS that were verified by clinical diagnosis, while unverified represents pathogens detected that were not supported by clinical diagnosis

This study was approved by the Ethics Committee of the First Affiliated Hospital of Xi’an Jiaotong University (Approval No. XJTU1AF2022LSK-242) and conducted in accordance with the Declaration of Helsinki. Informed consents of the patients were waived.

### Clinical data collection

Baseline characteristics of age and gender of enrolled patients were collected. In addition, time spent on diagnosis, length of antibiotic administration and length of hospitalization were recorded. The concordance of diagnostic results with clinical judgment for each diagnostic method, the type, consumption and the cost of antibiotics to the patient were collected as well.

### Culture and other conventional microbiological tests

In this study, culture-based methods are performed using all types of samples, such as blood, bronchoalveolar lavage fluid, sputum and secretions.

In addition, other conventional microbiological tests (CMTs) were also applied to detect pathogens in this study. The β-d-glucan (G) test (> 60 pg/mL, Genobio, Beijing, China) and galactomannan (GM) test (> 0.5 µg/L, Genobio, Beijing, China) were used to detect the invasive fungus infection. T-SPOT (QIAGEN, Hilden, Germany) was used for the detection of *Mycobacterium tuberculosis*. In addition, toxoplasmosis, rubella, cytomegalovirus, herpes simplex virus and Epstein-Barr virus (YHLO, Shenzhen, China) were detected by testing the specific IgM and/or IgG as needed. Other conventional diagnostic methods including blood smear, latex agglutination test, serologic tests, and nucleic acid amplification testing (traditional PCR, Xpert MTB/RIF [QIAGEN, Hilden, Germany]) were also performed according to the clinical needs. A positive CMTs result is defined as at least one of the above diagnostic methods being positive in a single sample.

### mNGS and bioinformatics analysis

Samples such as blood, bone marrow, cerebrospinal fluid, BALF and sputum were collected from patients. Cell-free DNA was extracted using the QIAamp DNA Micro Kit (QIAGEN, Hilden, Germany), followed by QIAseq Ultralow Input Library Kit for Illumina (QIAGEN, Hilden, Germany), and library construction using the Qubit (Thermo Fisher Scientific, MA, USA) and Agilent 2100 Bioanalyzer (Agilent Technologies, Palo Alto, USA) were used for library quality control. Qualified libraries were sequenced on the Nextseq 550 platform (Illumina, San Diego, USA). For bioinformatics analysis, short, low-quality, low-complexity and adapter-contaminated reads were filtered out from the raw data. Human host DNA reads were removed by mapping to the human reference genome hg38. The residual sequencing data were mapped to the Microbial Genome Databases (http://ftp.ncbi.nlm.nih.gov/genomes/) by implementing the Burrows-Wheeler Aligner software. A microbe would be considered mNGS positive if its coverage ranked the top ten of its corresponding microbe group and was not detected in the negative control (sterile water) or the ratio of reads per million (RPM) sample to control was greater than 10. The mNGS result would be considered positive for M. tuberculosis and Cryptococcus if at least one unique read was mapped to species level and was not detected in the control, or the sample-to-control ratio of RPM was greater than 5. The datasets presented in this study can be found in National Genomics Data Center (https://www.cncb.ac.cn/), accession no PRJCA010729.

### Statistical analysis

Statistical analysis of the data was performed using GraphPad Prism software (GraphPad Software, San Diego, CA). Continuous variables were expressed as mean ± standard deviation (SD). Categorical variables were expressed as count of patients (proportion). The diagnostic performance of mNGS, culture, and CMTs for the final clinical diagnosis (reference standard) was evaluated by using sensitivity, specificity, positive predictive value (PPV), negative predictive value (NPV) and total conforming rate (TCR). TCR was calculated as (number of samples in which the pathogen detected by the method is consistent with clinical diagnosis + number of samples in which both the method and clinical diagnosis are non-infected) / total number of samples with a confirmed clinical diagnosis. We further divided the patients with confirmed clinical diagnosis into two groups: the earlier mNGS detection (Patients sampled for mNGS within 3 days of admission) vs. the later mNGS detection (Patients sampled for mNGS after 3 days of admission). Differences between groups were compared by t-test or chi-square test. P ≤ 0.05 was considered statistically significant.

## Results

### Participant characteristics and the etiology of FUO

A total of 149 patients with FUO were included in this study. The clinical characteristics of these patients are shown in Table 1. 64 (43.0%) of them were female, with an average age of 48.8 ± 17.5 years. Of all cases, 53.7% (80) were eventually diagnosed as infectious diseases, 32.9% (49) were diagnosed as non-infectious diseases, while another 13.4% (20) were uncertain.

**Table 1:** Characteristics of patients with fever of unknown origin (FUO)

	Overall (N = 149)	Infection(N = 80)	Non-infection (N = 49)	Unknown (N = 20)
Female, n(%)	64 (43.0%)	29 (45.3%)	25 (39.1%)	10 (15.6%)
Age, years	48.8+/-17.5	50.4+/- 16.6	43.5+/-18.6	54.2 +/-17.4
Length of hospital stay, days	15.5+/-8.3	15.8+/-8.7	15.2+/-7.4	15.4+/-9.3
Type of antibiotics	2.34+/-1.75	2.57+/-1.92	1.88+/-1.53	2.50+/-1.38
Duration of antibiotics treatment, days	11.1+/-8.7	12.6+/-9.2	8.8+/-7.4	10.9+/-8.8

^*^Age, length of hospitalization, duration of antibiotics treatment were expressed as means+/-standard deviations (SDs) and gender was reported as the number of patients (proportions);

**Fig. 3:**
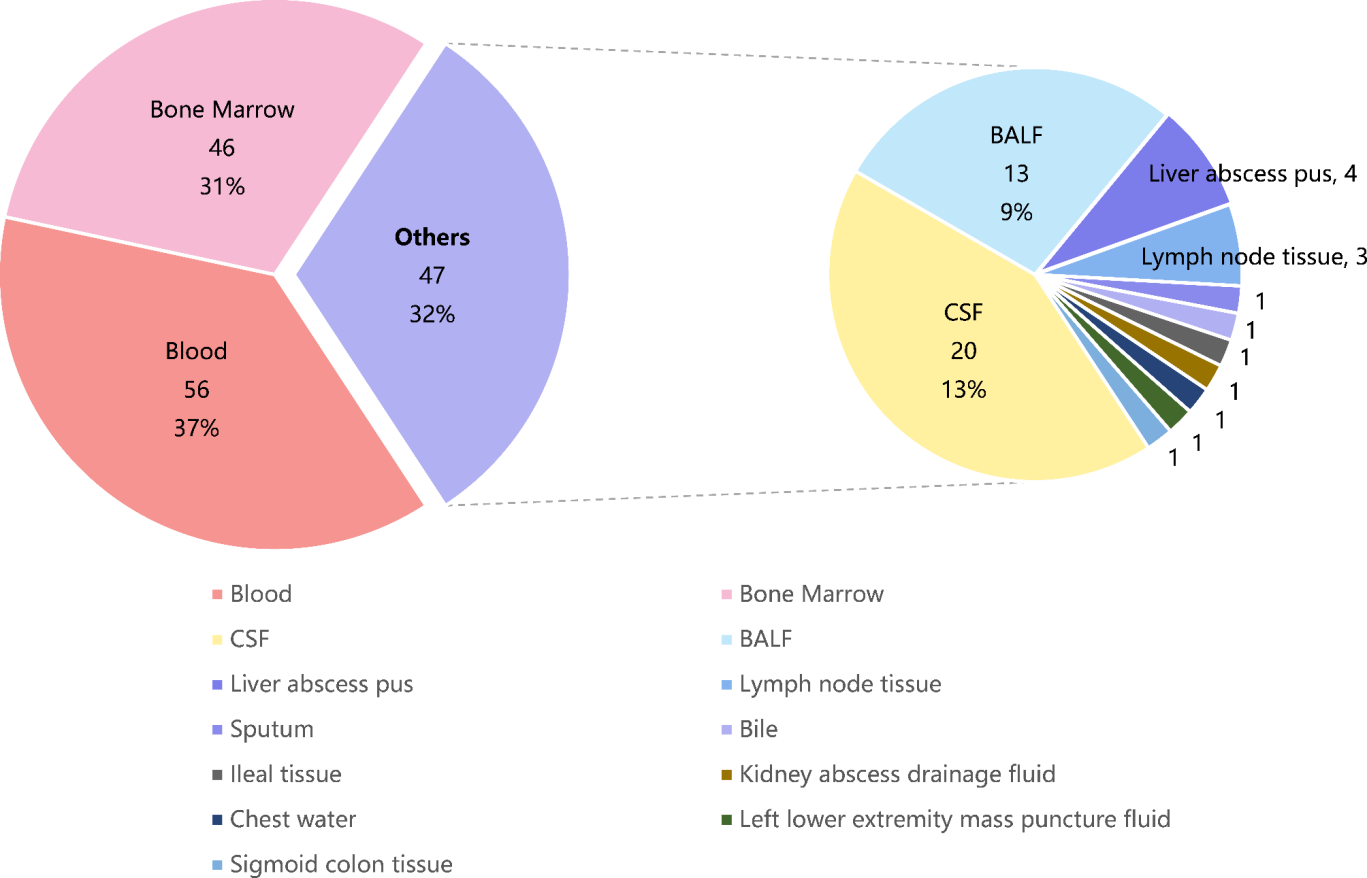
Types of samples used for pathogen detection

Specifically, in terms of diagnosed diseases (Table 2), the most common types of infectious diseases were respiratory infections (28.8%), bloodstream infections (25.0%) and central nervous system infections (18.7%), while the most common non-infectious diseases were NIID (52.8%) and tumors (13.2%).

**Table 2:** Types of diseases finally diagnosed

Disease	No.	Disease	No.	Disease	No.	Disease	No.	Disease	No.
Infection	80	NIID*	28	Tumor	7	Others	18	Unknown	20
Respiratory infections	23	Vasculitis	6	Lymphoma	2	Inflammatory response syndrome	9		
Bloodstream infections	20	Adult-onset Still’s disease	6	Multiple myeloma	1	Post-infection allergy	1		
Central nervous system infections	15	Haemophilic cell syndrome	5	Eosinophilia	1	Erythroderma type drug eruption	1		
Urinary infections	7	Arthrophlogosis	4	Leukemia	1	Necrotizing lymphadenitis	2		
Biliary duct infections	3	Connective tissue disease	1	Primary liver cancer	1	Erythema- -odosum	3		
Hepataposte–ma	7	Systemic lupus erythematosus	1	Myelodysplastic syndrome	1	Hypereosin- -ophilic dermatitis	1		
Nephrapostasis	2	Ulcerative colitis	1			Vaccination reaction	1		
Endocarditis	7	Chronic enteritis	1						
Abdominal infections	1	Polymyositis	1						
Lymphnoditis	2	Polyarteritis	1						
Mononucleosis	2	PBC	1						
Brucellosis	4								
Kala-azar	4								
Q fever	2								

*NIID: neuronal intranuclear inclusion disease

### Comparison of pathogen detection coverage of mNGS with that of culture and CMTs

To compare the pathogen detection coverage of mNGS and CMTs, the pathogen spectrum was calculated (Fig. 2). In total, mNGS detected 87 cases of bacterial pathogens, 23 cases of fungal pathogens, 74 cases of viral pathogens, and 6 cases of parasitic pathogens, compared to 60, 1, 11, and 1 cases detected by traditional methods, respectively. Among all pathogens detected by mNGS, the most detected bacteria were *Klebsiella pneumoniae* and *Escherichia coli*, the most detected fungi were *Candida albicans* and *Pneumocystis jirovecii*, and the most detected viral species were Human gammaherpesvirus 4 and Human betaherpesvirus 5.

The most detected pathogen species by culture and CMTs were similar, but the total number of detected species was much less than the number of species detected by mNGS. Many pathogenic organisms that were detected by mNGS and confirmed to be true positives were not detected by culture or CMTs. In particular, *Pneumocystis jirovecii* was detected five times in the mNGS detection and all were confirmed as true positives by final diagnosis, but was not detected by culture or CMTs at all.

To demonstrate more clearly the difference in pathogen detection coverage between methods, we calculated the detection of five commonly considered rare pathogens separately (Table S1). For the investigated rare pathogens, the overall detection rate of mNGS was 66.7%, compared to 0% for culture and 46.7% for CMTs. Such results suggest that mNGS could be more effective than traditional pathogen detection methods for the comprehensive detection of different types of pathogens, especially for rare pathogens.

### Diagnostic performance of mNGS compared to culture and CMTs

**Fig. 4:**
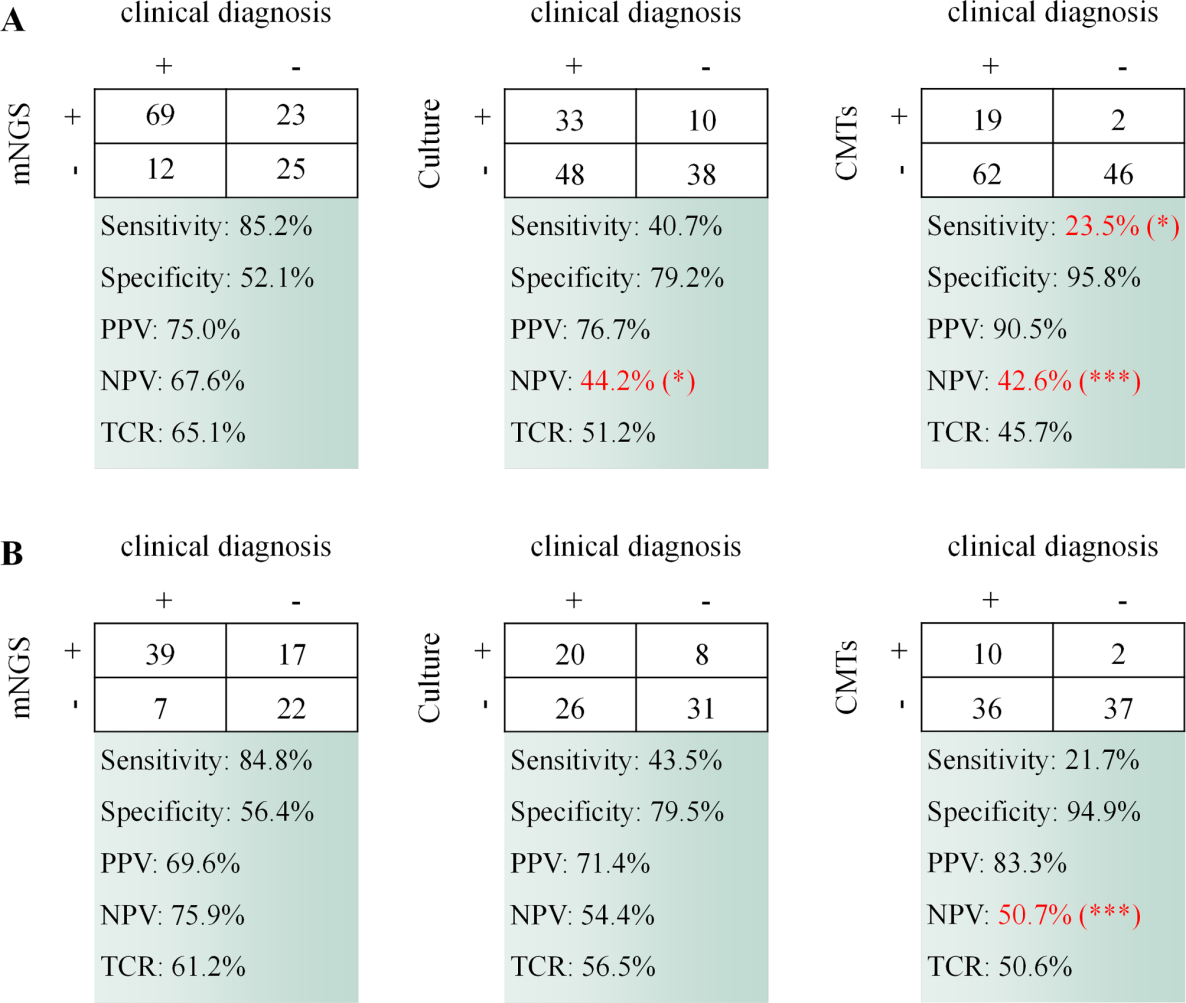
Comparison of the diagnostic performance of different methods. **(A)** Comparison of the diagnostic performance of the three methods in all samples. **(B)** Comparison of the diagnostic performance of the three methods in blood and bone marrow samples. Differences in significance between mNGS and culture, as well as mNGS and CMTs have been labeled. Significance was calculated by chi-square test. * represents p < 0.05, ** represents p < 0.01, and *** represents p < 0.001

Diagnostic performance of the three methods (mNGS, culture and CMTs) were investigated in all samples as well as in blood and bone marrow samples. The results showed (Fig. 4) that mNGS outperformed culture and CMTs in terms of sensitivity, negative predictive value (NPV) and total conforming rate (TCR), both for all samples and for blood and bone marrow samples. Of particular note, among all samples, the sensitivity of mNGS was 85.2%, significantly higher than that of CMTs at 23.5%. mNGS had an NPV of 67.6%, significantly higher than that of culture at 44.2% and CMTs at 42.6%. For blood and bone marrow samples, the NPV of mNGS was 75.9%, significantly higher than that of CMTs at 50.7%. However, it performed poorer in terms of specificity and positive predictive value (PPV).

This result shows that mNGS is an effective complement to traditional pathogen detection methods for different types of samples. In particular, its role in improving pathogen detection rates and excluding infections is evident.

### Impact of early mNGS detection on patient treatment and costs

**Fig. 5:**
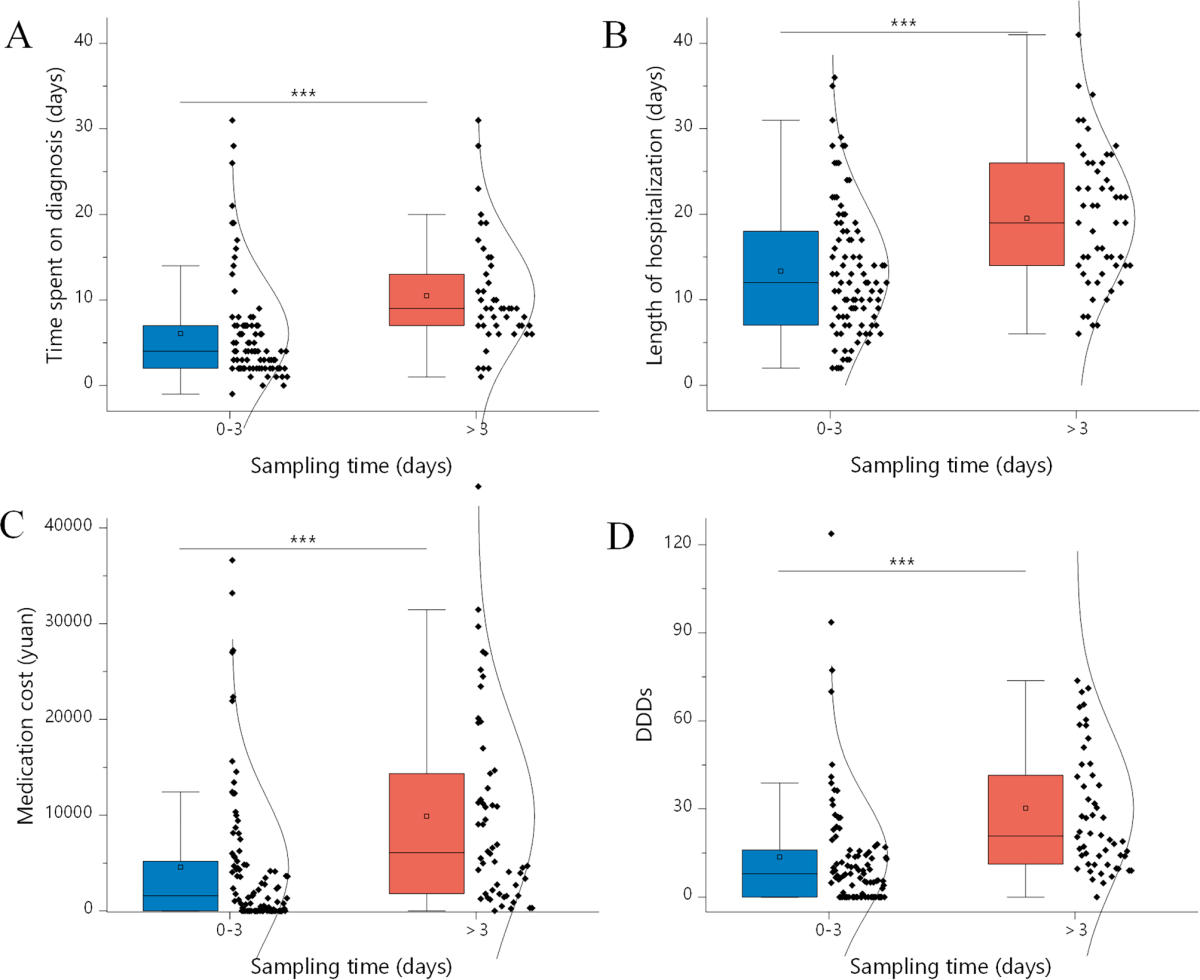
Effect of different sampling time for mNGS detection on **(A)** time spent on diagnosis, **(B)** length of hospitalization, **(C)** Medication costs per capita, and **(D)** defined daily doses (DDDs). * represents p < 0.05, ** represents p < 0.01, and *** represents p < 0.001

As a new technology that is just starting to be used in the clinic, there is a wide variation in the timing of when patients’ samples are collected for mNGS detection due to the patients’ varying acceptance of mNGS. Given that mNGS can be used as a complement to traditional detection methods, we further explored whether the earliness of its use for pathogen detection has an impact on the treatment and cost of FUO patients. Since day 3 after admission is generally considered to be an important time point for pathogen diagnosis [24], we retrospectively divided all patients into two groups: the earlier mNGS detection (sampling time: 0–3 days after admission to the department of infectious diseases) group and the later mNGS detection (sampling time: >3 days after admission to the department) group, and the results showed (Fig. 5) that earlier mNGS detection significantly reduced the time spent on diagnosis, length of hospitalization, Antibiotics Use Density (AUD), and medication costs per capita from the patients’ admission to the department of infectious diseases to discharge, although there is no remarkable difference in the pathogen spectrum between the two groups (data not shown).

## Discussion

Due to the complex etiology, FUO is a challenging disease that seriously bothers clinical internists [25]. In this study, in terms of etiologic classification, infection remained the main causative factor, with 53.7% (80) being various types of infectious diseases, including central nervous system infection, bloodstream infection, respiratory system infection, urinary system infection, biliary system infection, abscess, and infective endocarditis. Non-infectious diseases (including NIID, tumors, and other types) accounted for 32.88% (49/149), and their effective treatment also depended on the exclusion of the possibility of pathogenic infections. Therefore, for patients with FUO, obtaining accurate results of pathogen detection is crucial to definite diagnosis.

There are relatively few reports on the application of mNGS in FUO. Zhang et al. recommended that [24] the initiation of mNGS detection should be considered in conjunction with or in addition to the application of traditional techniques in patients presenting with fever or febrile syndrome, unknown etiology, and ineffective standardized empirical anti-infective therapy. Fu Zhangfan et al. suggested that [26] the use of mNGS in blood as a first-line investigation and the identification of samples from suspected infection sites as a second-line investigation in patients with FUO caused by infection could improve the overall diagnostic rate and serve as a promising option for optimal diagnosis.

In this study, we retrospectively analyzed clinical data from 149 FUO patients and found that mNGS not only detects pathogens in large numbers, but also provides a greater coverage of pathogen detection than traditional assays. Certain pathogens are known to be difficult to culture: not only do they require high media requirements, but also they require longer growth times [27, 28]. For example, *Mycobacterium tuberculosis* grows slowly, often taking 2–4 weeks or even up to 8 weeks to result in culture results [29], and *Brucella* usually takes at least 4 weeks [30]. This delays the clinicians’ decision making significantly. In contrast, because mNGS does not require culture, but rather sequencing and analysis of the entire genome of organisms in the specimen to obtain results for as many potential pathogens as possible, it expands the scope of detection [31] and provides a unique advantage to diagnose pathogens in FUO patients. In addition, it can obtain results within 24 h after sampling, greatly reducing the detection time. In this study, the detection rate of mNGS for rare pathogens was 66.7%, compared to 46.7% for culture and CMTs. mNGS clearly provides a faster and more effective method for detecting rare and uncommon pathogens.

Compared to traditional pathogen diagnostic methods, mNGS is slightly less specific, but has improved sensitivity, NPV and TCR, and is suitable for a wide range of sample types. To our knowledge, this study reports for the first time in a clinical study of FUO patients that mNGS has a significant advantage over traditional pathogen detection methods in terms of NPV. And this result implies that mNGS could be particularly suitable for ruling out infections. Diagnosis is often more difficult for non-infection-associated FUO, especially non-infectious inflammatory diseases. For example, Adult Still’s disease is an exclusionary disease [32] that requires exclusion of infection, lymphoma, etc. In clinical work, it is sometimes very difficult to achieve complete exclusion of infections due to the limitations of traditional pathogen testing [33]. In contrast, our results confirm that the comprehensiveness, high sensitivity and unbiased character of mNGS are indeed of great value in ruling out infections in FUO and deserve wide clinical application. In addition, the types of conventional diagnostic tests ordered very much depend on the knowledge and experience of the physician, which may vary between general practitioners and microbiologists, which could lead to the omission of potential pathogens such as M. tuberculosis or fungal pathogens that require specific tests (e.g., specific culture media) for detection. Therefore, mNGS supplements conventional diagnosis by providing physician with an overview of microbial composition of the clinical specimen(s) for correlation with patient’s clinical picture.

Clinicians need to make a correct diagnosis of any disease as early as possible, and in order to obtain diagnostic clues, rapid and accurate detection tools, in addition to detailed history taking and physical examination, can be of great help [27, 34]. In this study, we found that early mNGS detection in FUO patients can significantly reduce the time required for diagnosis and the length of hospital stay, which is beneficial to patients.

In addition, early mNGS detection can reduce the consumption and cost of antibiotics, which clearly facilitates the rational use of antibiotics. This is of great importance in clinical practice because it is well known that the irrational application of antibiotics has led to serious resistance problems and even the emergence of super drug-resistant bacteria [35], which are a serious threat to human health [36]. In the treatment of patients with FUO, especially for those without any localization clues, the empirical application of broad-spectrum antibiotics [37] is generally required due to the nature of their suspected infection without a clear pathogen. For some non-infectious inflammatory diseases, a full course of antibiotics is usually required for clinical judgment [38], which further exacerbates the misuse of antibiotics in such patients and leads to a number of problems such as pathogen resistance, secondary infection and dysbiosis in patients [36]. Early identification of the etiology allows for timely conversion of empirical treatment to targeted therapy, reducing the application of unnecessary comprehensive coverage; and early exclusion of infection allows for timely discontinuation of antibiotic therapy, ultimately benefiting patients and the public. Thus, although cost has been a concern regarding the use of mNGS in the diagnosis of infection, the cost per sample was 3000 renminbi [RMB] in 2019 (approximately $400) [39], mNGS would still be a meaningful complement to traditional tests with the advantages discussed above.

This study is a single-center retrospective study with limited number of patients enrolled. A multicenter prospective study is needed as a next step to further evaluate the value of mNGS in clinical applications.

In conclusion, mNGS could significantly improve the detected pathogen spectrum and clinical conforming rate of pathogens, especially rare pathogens, while having good negative predictive value for ruling out infections. Early mNGS detection may shorten the diagnosis time, hospitalization days and reduce unnecessary consumption of antibiotics. It has a broad application prospect in the pathogenic diagnosis of FUO.

## Electronic supplementary material

Below is the link to the electronic supplementary material.

Supplementary Material 1

## Data Availability

The datasets presented in this study can be found in National Genomics Data Center (https://ngdc.cncb.ac.cn/omix/release/OMIX001369).

## Abbreviations

mNGS: Metagenomic next-generation sequencing
FUO: Fever of unknown origin
NIID: Non-infectious inflammatory diseases
CMTs: Other conventional microbiological tests
GM test: Galactomannan test
PPV: Positive predictive value
NPV: Negative predictive value
TCR: Total conforming rate
AUD: Antibiotics Use Density
