# Supplementary material for: Early application of metagenomics next-generation sequencing may significantly reduce unnecessary consumption of antibiotics in patients with fever of unknown origin
Source: BMC Infect Dis. 2023 Jul 18;23:478. doi: 10.1186/s12879-023-08417-3 (PMC10354914; doi:10.1186/s12879-023-08417-3)
Supplement: Supplementary file 1 — Supplementary Material 1 [file 12879_2023_8417_MOESM1_ESM.docx]

**Supplementary materials**

**Tables**

Table S1 Comparison of detection rates of different methods for rare pathogens

|  | **mNGS** | **Culture** | **CMTs** |
| --- | --- | --- | --- |
| **Overall** | 66.7% (10/15) | 0% (0/15) | 46.7% (7/15) |
| **Tuberculosis** | 75% (3/4) | 0% (0/4) | 75% (3/4) |
| **Black fever** | 100% (4/4) | 0% (0/4) | 0% (0/4) |
| **Amoebiasis** | 100% (1/1) | 0% (0/1) | 100% (1/1) |
| **Brucellosis** | 0% (0/4) | 0% (0/4) | 75% (3/4) |
| **Q Fever** | 100% (2/2) | 0% (0/2) | 0% (0/2) |
